# “H” sprayer effect on liquid deposition on cucumber leaves and powdery mildew prevention in the shed

**DOI:** 10.3389/fpls.2023.1175939

**Published:** 2023-05-10

**Authors:** Weicai Qin, Xuan Chen, Panyang Chen

**Affiliations:** ^1^ Suzhou Polytechnic Institute of Agriculture, Suzhou, China; ^2^ Nanjing Institute of Technology, Nanjing, China

**Keywords:** H-Sprayer, fan nozzles, amount of deposition, shed, cucumber leaves, effectiveness of powdery mildew prevention

## Abstract

To clarify the effect of droplet size on solution deposition and powdery mildew control on greenhouse cucumber leaves, the effect of volume median droplet diameter (VMD) on solution deposition and maximum retention, as well as the effect of flusilazole on powdery mildew control on cucumber, was determined using the stem and leaf spray method. The VMD of the typical fan nozzles (F110-01, F110-015, F110-02, F110-03) of the selected US Tee jet production differs by approximately 90 μm. The results showed that the deposition of flusilazole solution on cucumber leaves decreased as the VMD of the droplets increased and that the deposition of the solution in the treatments with VMD of 120, 172, and 210 μm decreased by 22.02%, 10.37%, and 46. 97%, respectively, compared to that observed with treatment with 151 μm VMD. The deposition of the solution on cucumber leaves showed the highest deposition efficiency of 63.3% when the applied solution volume was 320 L/hm^2^, and the maximum stable retention of the liquid on the leaves was 6.6 µl/cm^2^. The control effects of different concentrations of flusilazole solution on cucumber powdery mildew differed significantly, and the best control effect was achieved at the dosage of 90 g/hm^2^ of the active ingredient, which was 15%−25% higher than that observed at the dosage of 50 and 70 g/hm^2^ of the active ingredient per hectare. A significant difference in the effect of droplet size on the control of cucumber powdery mildew was observed at any specific liquid concentration. Nozzle F110-01 showed the best control effect when the dosage of the active ingredient was 50 and 70 g/hm^2^ per hectare, which did not differ significantly from that observed with nozzle F110-015 but differed significantly from those observed with nozzles F110-02 and F110-03. Hence, we concluded that the use of smaller droplets with VMD of 100−150 μm, i.e. the choice of F110-01 or F110-015 nozzles, for application on the leaf parts of cucumber in the greenhouse under conditions of high liquid concentration, can significantly improve the effective use of pharmaceuticals and the disease control effect.

## 1 Introduction

Cucumber powdery mildew is an important disease caused by the monocotyledonous powdery mildew fungus, *Sphaerotheca fuliginea*, which is characterized by a short incubation period, frequent reinfestation ability, and high prevalence. The pathogen can infect cucumbers throughout the reproductive season (Hamza et al., 2015). Climatic conditions are the main factors influencing the occurrence of powdery mildew (Sarhan et al., 2020. Wu et al., 2022). Powdery mildew damage has increased with the development of protected vegetables. Currently, research on plant breeding for developing disease resistance and the spraying of pesticides are the two main ways of controlling this disease (Lv et al., 2016). The former is one of the popular methods used to prevent and control powdery mildew in many countries, and several disease-resistant varieties, such as Jin Za No. 1, have been developed (Tang, 2002). However, powdery mildew species may vary with countries or regions within the same country (Elad et al., 2021). In addition, the complexity and variability of the pathogen and the difficulty in identifying it accurately (Cerkauskas and Ferguson, 2014. Wang et al., 2021) render breeding for disease resistance challenging; this has hindered efforts to effectively address the occurrence of powdery mildew, which is controlled using pharmaceuticals.

As mentioned above, excessive use of pesticides can cause a certain amount of environmental pollution (Lan et al., 2018), and insufficient use can induce resistance (Tang et al., 2019. Elsharkawy et al., 2014). In addition to the selection of suitable agents, application techniques are equally important for the control of cucumber powdery mildew. In China, research on efficient application techniques for pest and disease control using different droplet sizes is limited, and easy-to-use spray quality standards are lacking, which makes blind operations common. In fact, the concentration of the liquid spray and the density of the droplets considerably affect the control of pests and diseases (Ye et al., 2022. Mao et al., 2022). Cui et al. (2010) investigated the effect of liquid application volume and droplet density on the control of wheat aphids, and the results showed that reducing the liquid application volume and increasing the liquid concentration did not only reduce the deposition volume but also considerably decreased the rate of pesticide loss; in addition, a certain density of the deposited imidacloprid droplet effectively controlled aphids in the wheat spike. Yang et al. (2012) studied the effect of nozzle type on droplet deposition and control of wheat aphids; to this end, they measured the performance of nozzles in terms of deposition volume, deposition uniformity, and ground loss rate to screen nozzles suitable for wheat field spraying. Hong et al. (2022) investigated the effect of UAV operating parameters on the spraying effect in a horsetail pine forest. Santos-Júnior et al. (2022) investigated the relationship between pesticide application rates on spray deposition, stink bug control, and soybean yield in soybean crops, and the results confirmed that pesticide sprays require a certain amount of droplet deposition and deposition density to be effective. Furthermore, as diseases caused by plant pathogens differ from damage caused by insect pests, different droplet sizes and application volumes are required (Eskandari and Sharifnabi, 2020).

Therefore, in this study, four typical fan nozzles with different apertures were used to investigate the effect of droplet size and the volume of different concentrations of pesticide on the control of powdery mildew on greenhouse cucumbers. Our observations will provide a basis for nozzle selection and the development of standards.

## 2 Material and methods

### 2.1 Materials

#### 2.1.1 Test cucumber

The test plant variety was Jimei Fuxing.

#### 2.1.2 Agents and instruments

The agents used are a 40% flusilazole emulsifier (Tianjin Sprela Pesticide Technology Development Co. Ltd. Tianjin, China) and a 1-g/L aqueous solution of rhodamine-B fluorescent tracer (Shanghai Qianduan Biological Co. Ltd. Shanghai, China). The instruments used include the following: an F95 fluorescence spectrophotometer (Shanghai Prismatic Technology Co. Ltd. Shanghai, China), a 2 cm in diameter hole puncher, a Spray Tec Fog Droplet Sizer (Shandong Necht Analytical Instruments Co. Ltd. Shangdong, China), and fan nozzles Tee jet F110-01, F110-015, F110-02, and F110-03.

#### 2.1.3 Test equipment

**Figure 1:**
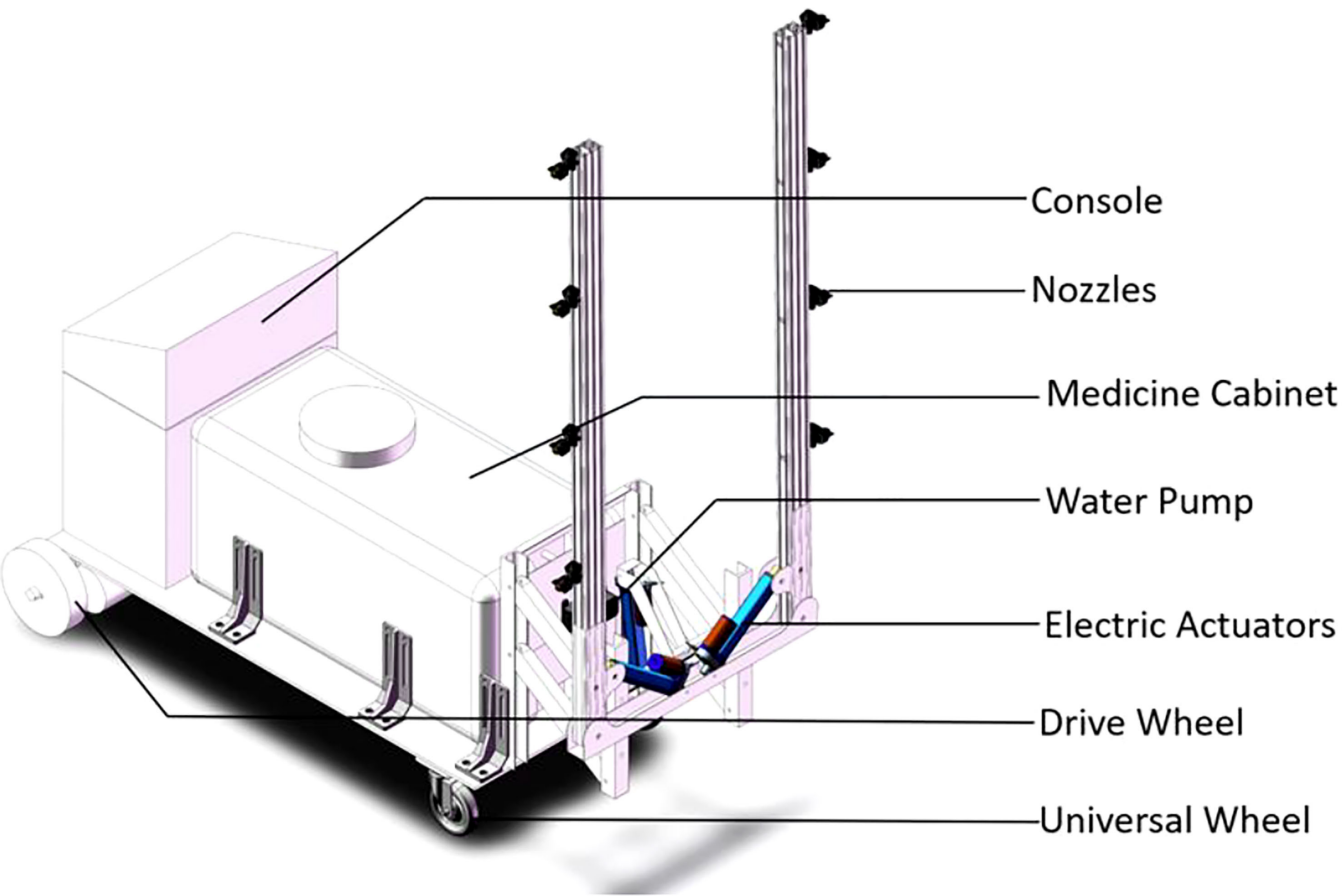
“H” type orbital stand crop sprayer. The sprayer was equipped with an adjustable pressure system and was operated at a spray pressure of 0.3 MPa to apply flusilazole for controlling powdery mildew on greenhouse cucumbers.

The test apparatus was an “H” type sprayer, as shown in **Figure 1**; the pressure was adjustable, and a spray pressure of 0.3 MPa was used. The detailed parameters are shown in **Table 1**.

**Table 1:** Parameters of “H” type sprayer.

Parameter	Numerical value	Remark
Dimension (mm)	1,603 * 777 * 1,806 (bracket vertical)	Length * width * height
Maximum chemical load (L)	400	Maximum capacity of the tank
Number of nozzles (pc)	8	Self-loading with different numbers of nozzles
Working height	0.3−1.8 m	Can be set on demand
Nozzle flow	7 L/min	Fan-shaped nozzle

### 2.2 Methods

#### 2.2.1. Nozzle selection and droplet size determination

Different models of fan nozzles, Tee jet F110-01, F110-015, F110-02, and F110-03, all with a 110° spray angle, were used. At a spray pressure of 0.3 MPa, the volume median diameter (VMD) of the droplets from different spray nozzles was measured using a Spray Tec Fog Droplet Sizer meter at a distance of 0.5 m from the spray nozzle. In total, 20–40,000 droplets were collected; the droplet VMD of the F110-01, F110-015, F110-02, and F110-03 spray nozzles were 120, 151, 172, and 210 μm, respectively. Single-nozzle flow rates of 390, 520, 820, and 1,200 ml/min, respectively, were used for the above spray nozzles.

#### 2.2.2 Effect of droplet size on the amount of flusilazole deposited

**Figure 2:**
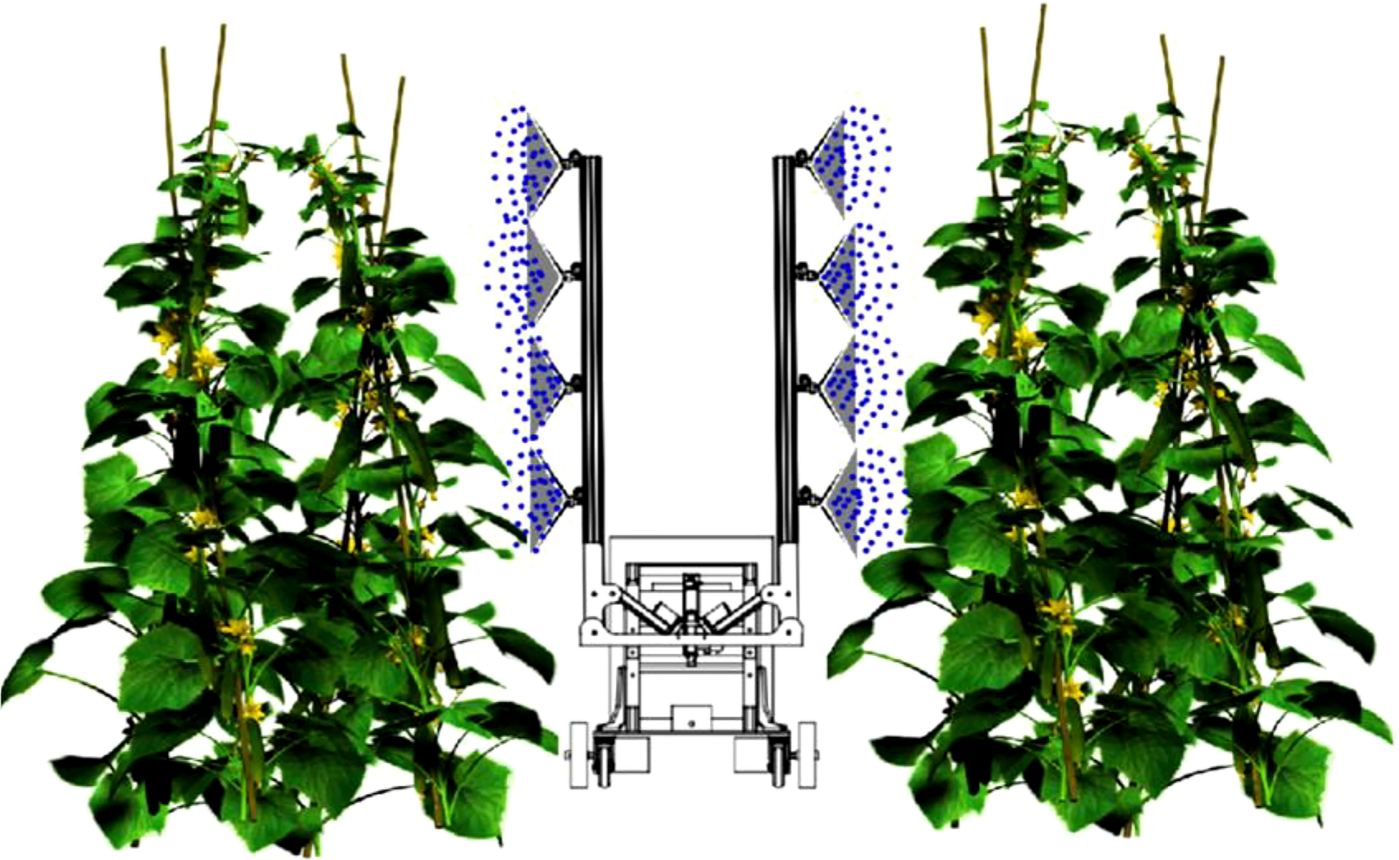
Diagram showing the spraying process used in the experiment, which was conducted on greenhouse cucumbers at the experimental base of Suzhou Polytechnic Institute of Agriculture. The experiment aimed to test the efficacy of four different VMD nozzles for controlling cucumber powdery mildew.

The experiment was performed on greenhouse cucumbers at the experimental base of Suzhou Polytechnic Institute of Agriculture, with plant spacing of 30 cm and row spacing of 50 and 90 cm. Control of cucumber powdery mildew (*S. fuliginea* (Schlecht) Poll). The test was conducted when the cucumbers grew to the 10−12-leaf stage, and cucumbers with uniform growth and powdery mildew characteristics were selected for the test. Four different VMD nozzles were used for the test; each nozzle was used to spray a plot of 10 m^2^, each plot was randomly arranged, and each treatment was repeated three times. Using tap water, prepare a mother solution of flusilazole with a concentration of 500 mg/L and a mother solution of rhodamine-B tracer with a mass fraction of 1%. In a 1:1 volume ratio, mix the rhodamine-B tracer mother solution and the flusilazole mother solution to prepare a mixed mother solution. The mixed mother solution was then used to prepare a medicine solution for spray treatment with a flusilazole concentration of 50 mg/L, and the spraying time was adjusted according to the flow rate of the nozzle such that the applied liquid volume of each treatment was 450 L/hm^2^ and the effective dose of flusilazole was 22.5 g/hm^2^. The spray pressure of the sprayer was set to 0.3 MPa. Five cucumber seedlings were randomly selected from each treatment after spraying, and each cucumber was marked from top to bottom by counting the second, fourth, seventh, and ninth leaves, which were used to measure the amount of flusilazole deposited per unit leaf area and calculate the average value. The spraying process is shown in **Figure 2**.

After the spray treatment, five holes were punched on the marked leaves using a hole puncher, placed in a self-sealing bag, brought back to the room, and eluted with 30 ml deionized water for 20 min. The absorbance value was measured after placing the eluate into a clean test tube at 572 nm. The amount of rhodamine-B in the eluate can be calculated from the standard curve of rhodamine-B. In this way, the amount of deposition of the spray liquid solution on the unit area can be calculated (Xue et al., 2014).The equation for calculating the amount deposited is as follows:


$$\beta_{dep}=\frac{\left(\rho_{smpl}-\rho_{blk}\right)\times F_{cal}\times V_{dii}}{\rho_{spray}\times A_{col}}$$

where $\beta_{dep}$ is the amount of mist droplet deposition in micrograms per square centimeter; $\rho_{smpl}$ is the fluorometer reading of the sample sampler; $\rho_{blk}$ is the fluorometer reading of the blank sampler (sampler + dilution water); $F_{cal}$ is the calibration factor (equal to the logarithm of the recovery rate) in micrograms per liter; $V_{dii}$ is the amount of diluent (e.g., tap water or deionized water) in which the tracer from the sampler is dissolved in liters; $\rho_{spray}$ is the concentration of the tracer in the spray solution in grams per liter; and $A_{col}$ is the area of the sampler in square centimeters.

#### 2.2.3 Maximum stable retention of flusilazole solution on cucumber leaves

The experiment was performed on greenhouse cucumbers at the experimental base of Suzhou Polytechnic Institute of Agriculture. The F110-015 spray nozzle was selected, and the spray pressure was 0.3 MPa. When the cucumber grew to the 10−12-leaf stage, 50 mg/L of flusilazole was sprayed at 110, 185, 320, 720, 950, and 1,100 L/hm^2^ dosages. Five cucumber seedlings were selected for each treatment, and each treatment was repeated thrice to determine the amount of flusilazole deposited per unit leaf area. The method was the same as described in **Effect of droplet size on the amount of flusilazole deposited**. The amount deposited on the leaves and the deposition efficiency, as well as the maximum stable retention of the solution on the leaves, i.e., the amount deposited when the amount of solution applied increases until there is no more droplet flow on the leaves, were calculated. The deposition efficiency was calculated as follows:


$$DE=\frac{D}{A}\times 100\%$$

where D is the amount of flusilazole deposited on the leaves (mg/m²) and A is the amount of flusilazole applied per unit area (mg/m²).

In total, 10 Petri dishes of 10-cm diameter were used for each treatment to collect the solution on the ground. The amount of solution applied per unit area is the ratio of the amount of solution collected to the area of the Petri dish.

#### 2.2.4 Effect of flusilazole solution on the control of cucumber powdery mildew

The experiment was conducted on greenhouse cucumbers at the experimental base of the Suzhou Polytechnic Institute of Agriculture. F110-01, F110-015, F110-02, and F110-03 spray nozzles were selected, corresponding to dosages of 110, 185, 320, and 500 L/hm^2^, respectively. The active ingredient dosages of flusilazole were 50, 70, and 90 g/hm^2^; 12 groups of treatments, one plot per treatment, and 10 m^2^ per plot were used; each plot was arranged randomly, and each treatment was repeated thrice. When cucumbers grew to the 10−12-leaf stage, the whole plant was sprayed with 50 mg/L of flusilazole, and blank control (CK) was set without spraying the pesticide. At the beginning of the disease, flusilazole was applied thrice at an interval of 7 days. Four random samples were taken from each plot, and all the leaves of the two plants were surveyed at each point. Grading was performed based on the area of the diseased spot on each leaf as a percentage of the whole leaf area. The significance of the control effect was determined based on Duncan’s new repeated difference (DMRT) method. Cucumber powdery mildew grading standard is as follows: grade 0: no disease spot; grade 1: disease spot area accounted for less than 5% of the whole leaf area; grade 3: accounted for 6%~10% of the whole leaf area; grade 5: accounted for 11%~20% of the whole leaf area; grade 7: accounted for 21%~40% of the whole leaf area; and grade 9: accounted for more than 40% of the whole leaf area. Disease development was investigated 10 days after administering the third dose, and the disease index and control effect (%) were calculated.


$$\text{DI}=\frac{\sum_{i}N_{i}\times W_{i}}{\text{T}\times 9}\times 100$$

where DI is the disease index, *N*_*i*_ is the number of leaves at level I, *W*_*i*_ is the relative level value of leaf level *i* (a decimal number between 0 and 1), *T* is the total number of leaves surveyed (a positive integer), and 9 is a constant, indicating the total number of leaf levels.


$$E=\frac{\text{lc}-\text{lp}}{\text{lc}}\times 100$$

where E is the effect of control (%), lc is the index of disease in the blank control area, representing the index of disease without any treatment, and lp is the index of disease in the treated area, representing the index of disease after specific treatment.

### 2.3 Data analysis

The SPSS 20.0 software was used for statistical analysis, and the Student–Newman–Keuls (SNK) method was applied to test the significance of differences. The analysis of variance (ANOVA) method was used to compare the differences between groups, and the SNK test was used for a two-way comparison between groups; *p* < 0.05 was considered significant.

## 3 Results

### 3.1 Effect of droplet size on the deposition of flusilazole solution on cucumber leaves

Droplet size is one of the most important parameters in spraying technology, and mastering the spray droplet size distribution is important for accurately controlling the spraying process (Kang et al., 2021). Droplets drift easily if their size is too small (less than 50 μm in diameter), whereas too large droplets (more than 450 μm in diameter) cannot penetrate the crop canopy. To solve this problem, Xu et al. (2015) proposed using larger droplets (narrow droplet spectrum with diameters larger than 140 μm) to overcome the drift and increase deposition.

**Figure 3:**
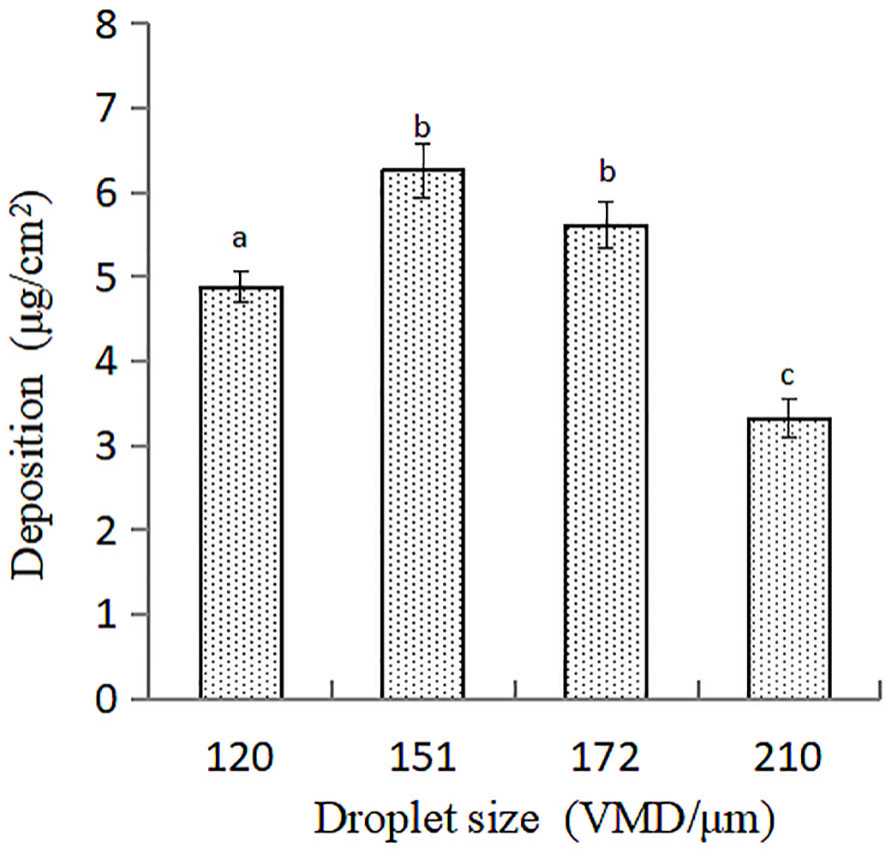
Deposition of flusilazole solution on cucumber leaves treated with different droplet sizes. The deposition was expressed as a volume of 50 mg/L flusilazole solution per cm^2^ of cucumber leaves. The spray volume used in each treatment was 450 L/hm^2^.

The amount of flusilazole solution deposited on cucumber leaves using different VMD mist droplet treatments differed significantly (**Figure 3**). The deposition of flusilazole solution was higher for treatment with droplets of 151 μm VMD and decreased with increasing VMD; compared to that observed with treatment with 151 μm VMD droplets, the deposition of flusilazole solution on cucumber leaves decreased by 22.02%, 10.37%, and 46.97% for 120, 172, and 210 μm VMD droplets, respectively.

### 3.2 Maximum stable retention of flusilazole solution on cucumber leaves

**Figure 4:**
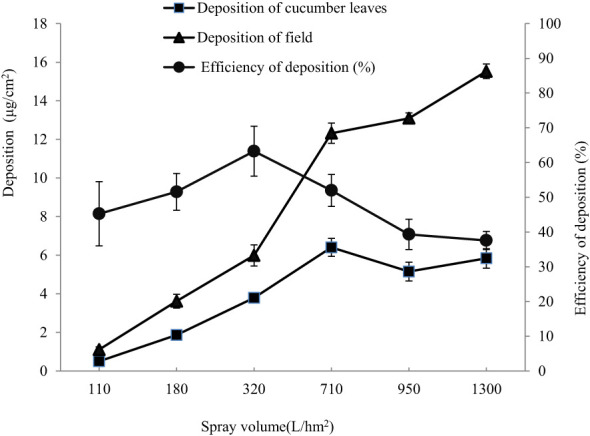
Maximum retention of flusilazole solution on cucumber leaves. The deposition was expressed as a volume of 50 mg/L flusilazole solution per cm^2^ of cucumber leaves. VMD of droplets was 151 μm.

The amount of flusilazole deposited on cucumber leaves varied with the amount of applied solution when treated with 50 mg/L of flusilazole solution (**Figure 4**). The deposition of flusilazole on cucumber leaves increased with the application rate when the latter was less than 720 L/hm^2^ and reached a larger value of 6.6 μl/cm^2^ when the application rate was 720 L/hm^2^.

The deposition on the leaves did not increase but rather decreased when the applied liquid volume exceeded 720 L/hm^2^. This indicated that the maximum stable retention of 50 mg/L of flusilazole solution on cucumber leaves was approximately 5.32 μl/cm^2^. A comparison of the amounts of flusilazole solution applied and deposited on cucumber leaves revealed that the deposition efficiency of flusilazole solution on cucumber leaves was relatively high when the amount of solution applied was less than 320 L/hm^2^, with about 63.3% of the flusilazole solution being deposited on cucumber leaves; when the amount of solution applied exceeded 320 L/hm^2^, the deposition efficiency decreased until the amount of solution applied exceeded 720 L/hm^2^, the deposition efficiency was 58.3%; following which it dropped sharply, the deposition efficiency was 37.6% after treatment with 1100 L/hm^2^ of the applied solution.

The deposition of field refers to the amount of pesticide solution that falls on plant leaves during the application process but is not absorbed and settles on the soil or ground below. According to the table data, it can be observed that as the amount of pesticide solution applied increases, the deposition of field also shows a gradually increasing trend. Within the range of pesticide solution applied, from 110 to 950, the deposition of field increased from 1.109 to 13.093, nearly 12 times. This means that at high levels of pesticide application, plants are unable to absorb all of the pesticides, leading to an increase in the amount of pesticide lost to the field and soil. Therefore, when applying pesticides, it is important to use appropriate amounts to reduce unnecessary drug loss and minimize environmental pollution risks.

### 3.3 Effect of flusilazole solution on the control of cucumber powdery mildew

Next, we compared the control effects of four fan spray nozzles, F110-01, F110-015, F110-02, and F110-03, with different pesticide dosages (active ingredient dosages of 50, 70, and 90 g/hm^2^) on mildew control. As shown in **Table 2**, using the same nozzle, an overall increasing trend in the control effect was observed as the amount of active ingredient applied increased. When the active ingredient dosage was 90 g/hm^2^, the lowest disease index of 2.25 was observed with the F110-01 nozzle, and its control effect was 89.48%, which was higher than the control effects of F110-015, F110-02, and F110-03. The control effects of F110-015, F110-02, and F110-03 were 87.61%, 86.06%, and 83.42%, respectively, which did not differ significantly; when the dosages of the active ingredient were 70 and 50 g/hm^2^, the control effect obtained upon using the F110-01 nozzle was 80.38% and 70.03%, respectively. The control effects of F110-015, F110-02, and F110-03 were higher than those of F110-015, F110-02, and F110-03. A significant difference was observed in the control effect of liquid concentration on cucumber powdery mildew; the disease index was significantly reduced at the active ingredient dosage of 90 g/hm^2^. The best control effect was 15%−25% higher than that observed with the active ingredient dosages of 50 and 70 g/hm^2^; the type of spray nozzle significantly affected the control effect. The control effect of F110-01 was the best at the same concentration, followed by those of F110-015, F110-02, and F110-03 (**Table 2**).

**Table 2:** Protective effect of flusilazole on cucumber powdery mildew (CPM).

Fungicides	Nozzles types	Dosage (g/hm^2^)	Disease index	Control efficiency (%; mean ± SD)
Flusilazole emulsion (40%)	F110-01	50	6.4 ± 0.21 bcde	70.03 ± 1.0 cd
		70	4.19 ± 0.28 efgh	80.38 ± 1.29 fg
		90	2.25 ± 0.10 h	89.48 ± 0.49 i
	F110-015	50	6.77 ± 0.38 bcd	68.32 ± 1.78 c
		70	5.43 ± 0.26 cdef	74.57 ± 1.22 de
		90	2.65 ± 0.12 gh	87.61 ± 0.58 hi
	F110-02	50	7.25 ± 05 bc	66.06 ± 2.34 c
		70	4.67 ± 0.45 defg	78.14 ± 2.09 ef
		90	2.98 ± 0.46 gh	86.06 ± 0.21 hi
	F110-03	50	8.1 ± 0.17 b	62.07 ± 0.78 b
		70	5.63 ± 0.3 cdef	73.64 ± 1.42 de
		90	3.54 ± 0.19 fgh	83.42 ± 0.89 gh
Blank control		–	21.37 ± 1.72 a	0.0 ± 0.0 a

The data in the table show mean ± SD. Data followed by different small letters in the same column differ significantly among different treatments at p < 0.05 level by Student–Newman–Keuls test.

**Table 3** shows that the effects of nozzle type and the amount of available active ingredient per hectare on the population were highly significant, with *p*-values less than 0.001. Furthermore, with the same type of nozzle, the control effect increased with the amount of active ingredient per hectare; considering nozzle F110-01 as an example, we observed a significant difference in the control effect with the amount of active ingredient; the control effect was 70.03%, 80.38%, and 89.48% with 50, 70, and 90 g/hm^2^ of active ingredient, respectively. As shown in **Table 2**, the type of nozzle significantly affected the control effect. At the same concentration, F110-01 showed the best control effect, followed by F110-01.5, F110-02, and F110-02.5.

**Table 3:** Inspection of effects among subjects.

Origin	Type III sums of squares	Df	Average square	F	Sig
Calibration model	3,536.742^a^	11	321.522	38.337	< 0.001
Intercept distance	285,177.501	1	285,177.501	34,003.204	< 0.001
Nozzle types	324.103	3	108.034	12.881	< 0.001
Active ingredient dosage	3,165.129	2	1,582.564	188.697	< 0.001
Error	301.924	36	8.387		
Total	289,016.167	48			
Total number of corrections	3,838.666	47			

Dependent variable: effectiveness of control.

a R^2 = ^0.921 (adjustment of R^2 = ^0.897).

## 4 Discussion

Although research on pesticide application technology in China has increased and the quality of the application equipment has been gradually improved, atomization of liquid chemicals, target plant collisions, and deposition of liquid on the target plants complicate the spray transfer process. Droplet movement to the blade surface (excluding needle leaves) due to kinetic energy, surface energy, and electrostatic energy and blade contact angle at the droplet-leaf interface jointly determine the droplet impact at the interface after adhesion, bounce, and spray (Williams et al., 2008). However, related basic research on the spraying technology still needs to be improved (Roisman et al., 2006). Some studies have shown that the angle of mist flow affects the deposition of pesticide solutions on target plants and that 20°−40° or 60° mist flow angles are best for the deposition of solutions on simulated upright targets (Dhalin et al., 2008).

As the cucumber leaf surface has many ridges, the critical surface tension value is relatively low, and its adhesive tension is 6.2 mN/m; hence, the mist droplets are easily lost (Feng et al., 2022). The size of the pesticide droplets deposited on the cucumber leaves considerably affects their effectiveness; coarse droplets formed during movement under the influence of gravity are relatively large, while bouncing off droplets from leaves after collision leads to considerable loss (Song et al., 2019).

Under conditions where personnel control the uniformity of spraying speed, such as high-capacity coarse fog spraying as well as liquid flow spraying, the spraying of large droplets evidently increases the amount of liquid applied; however, changing the pattern of pesticide use by small-scale individual farmers in China is difficult. The results of this study show that the effective use rate decreases significantly as the VMD of the spray droplets increases and the volume of the applied liquid increases. Spray quality depends largely on the spray nozzle; different pesticide atomization methods can produce droplets of varying sizes; however, for a particular organism or part of the organism, only a certain droplet size can be captured to produce an effective toxic effect. This was discovered in the 1950s, and after extensive research, it was gradually accepted and recognized by researchers (Mount, 1970; Johnstone, 1971; Harburguer et al., 2012). Studies have shown that the optimum particle size is 10−50 μm for flying pests, 30−150 μm for cotton bollworms, 30−150 μm for treating diseases, and 100−300 μm for weeds.

In this study, the amount of flusilazole deposited on cucumber leaves increased with increasing spray volume when the spray volume was less than 720 L/hm^2^, reaching a maximum of 6.6 μl/cm^2^ at 720 L/hm^2^. However, when the spray volume exceeded 720 L/hm^2^, the amount deposited on the leaves no longer increased but decreased. As a rule, the higher the spray volume, the greater the deposition will be. This is because more spray volume causes more liquid or particulate matter to fall on the target surface, increasing the amount of surface deposition. However, an increase in spray volume does not always increase the amount of deposition linearly. When the spray volume is very high, saturation may occur and the surface cannot absorb any more liquid or particulate matter, at which point increasing the spray volume will not increase the amount of deposition. Therefore, the spray volume should be controlled within an appropriate range to ensure optimum deposition of flusilazole solution on the leaves.

In addition, there was an overall trend toward increasing control effectiveness with increasing dosages of active ingredients. At the same dosage, sprayers with smaller droplet VMD had better control, while the lowest disease index and best control were achieved with F110-01 sprayers. There was also a significant difference in the control of cucumber powdery mildew due to the concentration of the solution. At an active ingredient dosage of 90 g/hm^2^, the disease index was significantly lower, and the control effect was the best, being 15%–25% higher than at 50 and 70 g/hm^2^. The type of spray nozzle also had a significant effect on the control effect. At the same concentration, F110-01 had the best control effect, followed by F110-015, F110-02, and, worst of all, F110-03. Therefore, for the control of powdery mildew, we can consider using the F110-01 nozzle with small droplet VMD and applying it at an active ingredient dosage of 90 g/hm^2^ per hectare to obtain the best control effect. At the same time, attention to the adjustment of the concentration of the liquid and the rational use of the spray nozzles are also important measures to improve the control effect.

## 5 Conclusion

In this study, the flusilazole water emulsion was applied to cucumber leaves at a uniform 45° angle to minimize the impact of the droplets on the leaf surface and thereby reduce the amount of solution lost *via* droplet bouncing. The deposition of flusilazole solution on greenhouse-grown cucumber leaves decreased with increasing VMD of the droplets (**Figure 4**), and the use of small droplets with a VMD of about 160−200 µm facilitated the deposition of the solution on cucumber leaves. Based on the results of a large number of experiments conducted using the blown mist method, we concluded that the deposition efficiency of the drug may be more desirable if the VMD of the mist droplets is further reduced, which requires further investigation.

The deposition efficiency of flusilazole solution on cucumber leaves was relatively high when the volume of the applied solution was less than 320 L/hm^2^; about 63.3% of the flusilazole solution was deposited on cucumber leaves, and a small amount was deposited on leaf stems, but nearly one-third of the solution was still lost to the soil; the deposition efficiency decreased further when the applied liquid volume exceeded 320 L/hm^2^. The deposition efficiency was 58.3% when the applied liquid volume exceeded 720 L/hm^2^, following which it decreased sharply to 37.6% when treated with an applied liquid volume of 1,100 L/hm^2^. This is similar to the observation of Lu et al. (2010), who observed that leaf retention started to increase rapidly with increasing application rate; after reaching the attrition point, the application rate further increased, while the retention rate decreased and gradually tended to a stable value, which was the maximum stable retention rate, indicating that the retention rate of leaf solution would not increase when the application rate exceeded the attrition point. During the cultivation of cucumber, the recommended amount of solution applied often reaches or even exceeds 720 L/hm^2^, resulting in low efficiency of liquid deposition, which is an important reason for the unsatisfactory control effect.

We observed a significant difference in the effect of the concentration of the solution on the control of cucumber powdery mildew, with the best effect observed at 90 g/hm^2^ of the active ingredient, which was 15%−25% more effective than those at 50 and 70 g/hm^2^ of the active ingredient per hectare. For a specific concentration of the liquid, a significant difference was observed in the effect of droplet size on the control of cucumber powdery mildew. When the amount of active ingredient was 50 and 70 g/hm^2^ per hectare, nozzle F110-01 showed the best control effect, which did not differ significantly from that observed with nozzle F110-015 but differed significantly from those observed with nozzles F110-02 and F110-03. This may be because the fine or medium particle size droplets were blocked less by the crop leaves, which is more conducive for the penetration of the droplets into the crop canopy; as a result, the droplets are deposited in the inner layer of the crop, achieving a better control effect. According to the results of this study, the use of smaller droplets with a VMD of 100−150 µm for application to the upper part of the leaves of cucumber in greenhouses, i.e., the use of F110-01 or F110-015 nozzles, can significantly improve the effective use of pharmaceuticals and the disease control effect.

We have only analyzed the effect of the spray hole diameter of different fan nozzles in this study; in the future, cone nozzles or other types of fan nozzles should be tested to reduce the amount of liquid applied and enhance the scientific use of drugs.

## Data availability statement

The original contributions presented in the study are included in the article/supplementary material. Further inquiries can be directed to the corresponding author.

## Author contributions

WQ, XC, and PC: Investigation, Writing-Original draft preparation. XC: Investigation. WQ: Conceptualization, Supervision, Writing- Reviewing and Editing. All authors contributed to the article and approved the submitted version.
